# Dietary Oils Alter Lipid and Volatile Compound Profiles of Donkey Milk: A Comprehensive Analysis

**DOI:** 10.1002/fsn3.70291

**Published:** 2025-05-20

**Authors:** Wei Ren, Lingyun Sun, Xinyi Du, Yile Chen, Yinghui Chen, Huili Liang, Xiyan Kou, Muhammad Zahoor Khan, Changfa Wang, Mengmeng Li

**Affiliations:** ^1^ School of Agriculture and Biology Liaocheng Research Institute of Donkey High‐Efficiency Breeding and Ecological Feeding, Liaocheng University Liaocheng China

**Keywords:** dietary oils, donkey milk, lipids, volatile compounds

## Abstract

Milk lipids and volatile compounds (VOCs) are critical parameters for evaluating milk quality. Through lipidomics and flavoromics assays, this study investigates the molecular characteristics of milk lipids and VOCs in donkeys fed with different oils. The results showed that 725 lipids from 21 subclasses were identified, including 57% triglyceride and 12% diglyceride. Furthermore, 110 differential lipids were detected, 90% of which contained unsaturated fatty acids. TG (18:2_13:0_18:2) was considered a potential marker to distinguish different donkey milk. These different lipids are involved in 20 metabolic pathways, with glycerophospholipid metabolism and glycerolipid metabolism being the most relevant. Additionally, 391 VOCs were identified, with heterocyclic compounds, terpenoids, and alcohols being the most prevalent. Seventy VOCs were differentially regulated in donkey milk, predominantly consisting of aldehydes, heterocyclic compounds, and terpenoids, which were more abundant in the palm oil group. Altogether, our study provides novel insights into how dietary oil supplementation alters the quality of donkey milk.

## Graphical abstract

More comprehensive lipids (725) and VOCs (391) were excavated in donkey milk. Dietary oils significantly affect the lipid and volatile compositions of donkey milk. The eight metabolism pathways, including glycerophospholipid metabolism and glycerolipid metabolism, are most relevant to lipid metabolism.

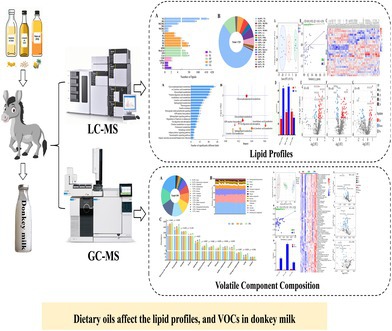

## 1 Introduction

Milk is the most widely consumed dairy product globally, serving as a primary source of beneficial bioactive components for consumers. Notably, donkey milk closely resembles human milk, with a nutritional composition that is 99% similar, thereby representing an important dietary component (Zhang et al. 2021). Donkey milk is rich in selenium, lysozyme, essential amino acids, vitamin C, functional whey protein, and polyunsaturated fatty acids (PUFAs), and it contains lower levels of fat and cholesterol compared to cow's milk (Chiofalo et al. 2011; Li et al. 2022; Lionetti et al. 2012). Lipids and volatile compounds (VOCs) are key quality indicators in dairy products. Lipids, as crucial components of cell membranes, play roles in energy provision, fat‐soluble vitamin transportation, and cell signaling (Souroullas et al. 2018; Vincenzetti et al. 2021; Yue et al. 2020). VOCs, recognized as significant contributors to consumer food choices, enhance the overall sensory quality of food (Clarke et al. 2022; Dan et al. 2019). Consequently, the manipulation of lipids and VOCs is important to improve the quality of donkey milk.

Milk lipids, as sources of bioactive lipids and energy, contribute to reducing inflammatory responses, preventing cardiovascular diseases, combating cancer, and supporting neural development (German and Dillard 2006; Jia et al. 2022; Trinchese et al. 2018). These compounds are influenced by various factors such as species, physiological condition, and nutrition (Liu et al. 2018). Dietary factors have been identified as the most significant influence on milk lipid content; for example, the triglyceride (TG) levels in donkey milk are significantly increased when the diet includes corn straw compared to wheat straw and wheat husk (Ren et al. 2023). The amount of C16:1 c‐9, C18:3 c‐9, c‐12, c‐15, and C18:1 t‐11 is positively correlated with the amount of the corresponding dietary fatty acid (FA) (Riuzzi et al. 2021). Furthermore, dietary oils can lead to the enhancement of milk quality by being transported into milk or converted into other biomolecules with nutraceutical properties (Steijns 2001). The inclusion of sunflower seed oil in the diet has been shown to enhance milk fat secretion and alter the composition of milk FAs (Bernard et al. 2009), and supplementation with oil seeds has resulted in increased levels of unsaturated 18‐carbon fatty acids (Plata‐Pérez et al. 2022). Thus, the lipid composition of donkey milk is significantly affected by dietary lipids, particularly oils.

Several VOCs, originating from the oxidation and hydrolysis of milk fat, primarily include free FAs, 2‐alkyl ketones, γ‐ and δ‐lactones, aldehydes, and ketones (Ho et al. 2015). The complex system of VOCs in milk was affected by the feed and internal metabolism of lactating mammals (Clarke et al. 2022). In ewes fed with a mixture of *lolium perenne* and *trifolium squarrosum* , rough pasture, and *avena sativa* , (E,E)‐3,7,11‐tri‐methyl‐2,4,10‐dodecatriene was detected as a marker of rough pasture by gas chromatography–mass spectrometry (GC–MS) (Povolo et al. 2007). Incorporating flaxseed into the diet of dairy cows significantly enhances the concentration of n‐3 PUFA in raw milk, which subsequently induces alterations in the profile of VOCs, including acids, esters, aldehydes, alcohols, and ketones (Huang et al. 2022). The content of linalool in breast milk was found to be significantly increased when consuming standard curry dishes (Debong et al. 2021). Several studies have found that the addition of pasture plant essential oils increased the levels of phenols, δ‐cadinene, 1,8‐cineole (eucalyptol), β‐phellandrene, and methyl salicylate in milk (Tornambé et al. 2008). The γ‐12:2 lactone exhibits significant odor activity in the milk of dairy cows fed a Total Mixed Ration diet, which consists of maize silage, grass silage, hay, whole cottonseed, maize grain, barley, soybean, fishmeal, vegetable oil, protected fat, corn gluten, molasses, minerals, and vitamins, compared to cows fed a pasture diet composed of ryegrass, white clover, *Poa annua* , and plantains (Bendall 2001). The flavor of milk was modified by feeding cows dried olive pomace, and especially the content of free fatty acids, ketones, lactones, esters, and phenylalanine catabolic products was significantly increased (Castellani et al. 2017). The milk of cows fed perennial ryegrass contained significantly higher levels of 1‐methylpropyl butyric acid, 1‐butanol, dodecanal, and butyl butyrate. In contrast, the milk of cows fed an all‐mixed ration was characterized by the presence of unique compounds, including 3‐methylbutyric acid, 2‐methylpropanal, and 2,5‐dimethylpyrazine (Clarke et al. 2022). However, the specific influence of feed oil type on milk flavor remains unclear and warrants further investigation.

This study aims to comprehensively identify and quantify lipids and VOCs in the milk of donkeys fed with various oils, employing liquid chromatography‐mass spectrometry (LC–MS) and GC–MS techniques. The findings are expected to offer novel insights and valuable information for assessing the nutritional value of donkey milk and facilitating the development of donkey milk products.

## 2 Materials and Methods

### 2.1 Animal Experiments

All animal experiments received approval from the Animal Care and Use Committee of Liaocheng University (2023022706). The study involved twenty‐seven healthy, four‐year‐old lactating female donkeys, which were procured from a local donkey herd in Shandong Province, China. These donkeys were meticulously selected based on uniformity in body weight, parity, and reproductive performance to ensure consistency in the study parameters. The donkeys were randomly assigned to three groups, with each group receiving a different basic dietary oil: soybean oil (SO), linseed oil (LO), and palm oil (PO). Furthermore, Table 1 delineates the formulation of three experimental diets alongside the FA compositions of the respective oils. Throughout the four‐week summer period, the donkeys were fed the diet twice daily, at 9:00 and 16:00. The experiment was conducted during the summer months of July and August (30°C ± 5°C). Following completion of the experimental phase, a 100 mL milk sample was collected from each donkey involved in the experiment. A portion of 50 mL from each milk sample was treated with preservatives (0.05 g potassium dichromate) and immediately transported to the laboratory for component analysis. Prior to conducting lipid and VOC analysis, the milk samples were subjected to freezing at −20°C and subsequently stored at −80°C. This stringent protocol was established to safeguard the preservation and integrity of the samples, thereby facilitating accurate analytical results.

**TABLE 1:** Ingredients, nutrient content and fatty acid of diets for donkeys.

Item	SO	LO	PO
Ingredients (%)			
Corn	14.71	14.71	14.71
Wheat bean	2.75	2.75	2.75
Soybean	1.76	1.76	1.76
Salt	0.20	0.20	0.20
Premix^1^	0.39	0.39	0.39
Wheat straw	80.00	80.00	80.00
Soybean oil	1.00	—	—
Linseed oil	—	1.00	—
Palm oil	—	—	1.00
Total	100	100	100
Proximate composition (% dry matter)			
Dry matter	94.37	93.94	94.18
Crude protein	6.48	6.39	6.65
Crude fat	4.09	4.06	4.17
Crude ash	10.32	10.64	10.14
Calcium	0.29	0.36	0.34
Phosphorus	0.15	0.13	0.18
Salt	0.66	0.67	0.63
Major fatty acid (% total fatty acid)			
C14:0	0.75	0.71	1.42
C16:0	16.51	15.05	23.30
C18:0	2.64	2.70	2.68
C18:1	19.21	18.75	20.58
C18:2n‐6	50.68	42.39	41.85
C18:3n‐3	2.47	14.52	2.55

*Note:* Ingredients, proximate composition, and major fatty acids were measured and analyzed based on triplicate determination. ^1^Premix/kg: vitamin A, 275 KIU; vitamin D_3_, 115 KIU; vitamin E > 180 IU; Fe (green vitriol), 3500 mg; Cu (copper sulfate), 725 mg; Zn (zinc sulfate), 2500 mg; Mn (manganese sulfate), 1750 mg; I (calcium iodide), 54 mg; Se (sodium selenite), 7.3 mg; Methionine ≥ 0.2%; Lysine ≥ 1%.

### 2.2 Milk Component Analysis

The main milk components (fat, protein, lactose, solids, solids‐not‐fat, freezing point depression, lactoferrin) were analyzed by DairySpec Combi (Bentley Instruments Inc., chaska, MN, USA). The NexGen system utilized a Fourier Transform Infrared (FT‐IR) spectroscopic instrument, and the sample was maintained at a temperature of 40°C within the instrument. The set points for the temperature controllers are as follows: Cell = 42°C, Homogenizer = 42°C, Reservoir = 42°C, Air = 35°C. Carrier Fluid (2% RBS) is used as a carrier fluid for the flow cytometer and as a cleaning solution in both instruments. The milk samples were preheated to 40°C before analysis. An automatic sampler was used with a stirring speed of 400 rpm for 4 s. Each sample was tested twice.

### 2.3 Lipid Profile Analysis by LC–MS

Lipids from milk were extracted by chloroform/methanol (2:1, *v*/*v*, pre‐cooled to −20°C). Chromatographic separation was achieved using a Shim‐pack UFLC SHIMADZU CBM30A UPLC system carried out on an ACQUITY UPLC BEH C18 (2.1 × 100 mm, 1.7 μm, Waters) column maintained at 50°C. Gradient elution of analytes was carried out with 60% acetonitrile +40% water (0.1% formic acid +10 mM ammonium formate) (A) and 90% isopropanol +10% acetonitrile (0.1% formic acid +10 Mm ammonium formate) (B) at a flow rate of 0.25 mL/min. Injection volume was 2 μL and the temperature of the autosampler was 8°C. An increasing linear gradient of solvent A (*v*/*v*) was used as follows: 0 ~ 5 min, 70 ~ 57% A; 5 ~ 5.1 min, 57% ~ 50% A; 5.1 ~ 14 min, 50% ~ 30% A; 14 ~ 14.1 min, 30% A; 14.1 ~ 21 min, 30% ~ 1% A; 21 ~ 24 min, 1% A; 24 ~ 24.1 min, 1% ~ 70% A; 24.1 ~ 28 min, 70% A. Electrospray ionization source (ESI) spray voltage was 3.5 kV (ESI‐, 2.5 kV). Data dependent acquisition MS/MS experiments were performed with HCD scan, and the normalized collision energy was 30 eV. The dynamic exclusion method was used to remove some unnecessary information from the MS/MS spectra. The lipids were annotated and aligned by LipidSearch (v4.0).

### 2.4 VOCs Analysis by GC–MS

The VOCs in donkey milk were extracted by a head space solid‐phase microextraction (HS‐SPME, 120 μm DVB/CWR/PDMS). The samples were shaken at 60°C for 5 min and then resolved at 250°C for 5 min. A GC (Agilent, 8890–7000D) equipped with a capillary column (DB‐5MS, 30 m × 0.25 mm × 0.25 μm, Agilent J&W Scientific, Folsom, CA, USA) was used to separate and characterize the VOCs in the samples. The rate of the helium carrier gas was 1.2 mL/min and injector temperature was 250°C. The mass spectrometer was operated in electron ionization mode, in selected ion detection mode combined with qualitative and quantitative ion precise scanning. The VOCs were identified by NIST mass spectral library and retention indices, and search software.

### 2.5 Statistical Analysis

The data analyses were conducted using SPSS v26.0 (SPSS Inc., Chicago, IL, USA) with ANOVA, Tukey's test, Duncan's multiple range test, and Fisher's protected least significant difference (LSD) test. The values were presented as mean ± standard error of the mean (SEM, *n* = 9), and significance was set at *p* < 0.05. Different lipids and VOCs were filtered with variable importance in projection (VIP) > 1 and *p* < 0.05, indicating significant differences. Different analyses, orthogonal partial least squares discriminant analysis (OPLS‐DA), heatmap analysis, and volcano plots were employed by majorbio online software and R software.

## 3 Results

### 3.1 Nutritional Composition of Donkey Milk

As presented in Table 2, the fat contents were higher in the PO group than those in the LO and SO groups. The contents of protein, solid, solid‐not‐fat, urea, freezing point depression (FPD) and lactoferrin were elevated in the SO group compared to the PO and LO groups. Conversely, the contents of SFA and PUFA were lower in the SO and LO groups than in the PO group. Notably, the LO group has the lowest level of SFA. In contrast, the PO group has higher levels of both SFA and PUFA compared to the other groups.

**TABLE 2:** Nutritional composition of donkey milk (%).

Item	SO	LO	PO	SEM	*p*
Fat	0.25^b^	0.07^a^	0.26^b^	0.018	0.000
Protein	1.81^c^	1.65^b^	1.38^a^	0.024	0.000
Lactose	6.59^a^	6.73^b^	6.80^c^	0.016	0.000
Solids	9.43^c^	9.22^b^	9.02^a^	0.040	0.000
SNF	9.23^b^	9.20^b^	8.92^a^	0.027	0.000
Urea	14.53^b^	15.43^b^	12.59^a^	0.332	0.002
FPD (°C)	457.84^b^	453.85^b^	443.60^a^	1.191	0.000
Lactoferrin	9.78^c^	9.53^b^	9.34^a^	0.043	0.000
SFA	0.15^ab^	0.08^a^	0.28^b^	0.013	0.007
PUFA	0.20^a^	0.20^a^	0.24^b^	0.005	0.003

Abbreviations: FPD, freezing point depression; PUFA, polyunsaturated fatty acid; SFA, saturated fatty acid; SNF, solids not fat. a‐b Means in the same row with different letters differ significantly, *p* < 0.05; ab Means in the same row with different letters do not differ significantly, *p* > 0.05.

### 3.2 Lipid Profiles of Donkey Milk

**FIGURE 1:**
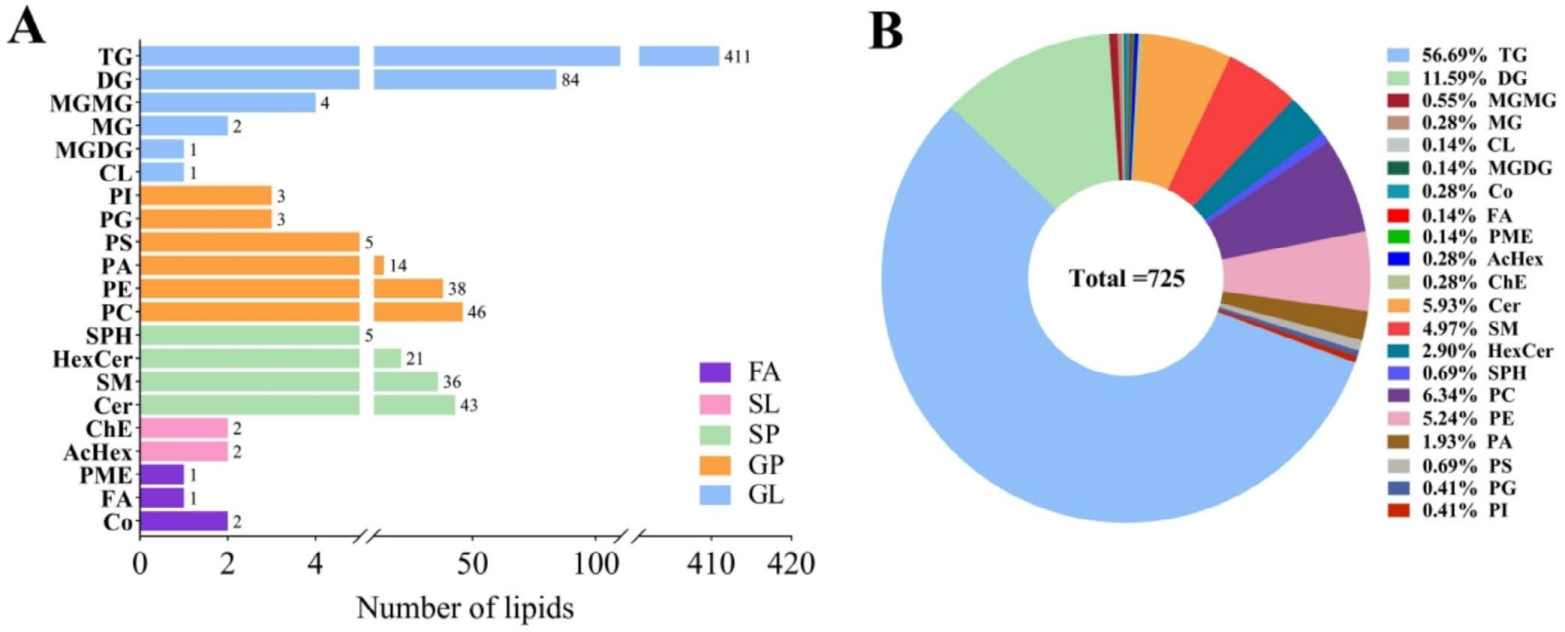
Qualitative analysis results of lipids in donkey milk. Numbers (A) and percentages (B) of lipid subclasses.

As shown in Figure 1, a total of 725 lipids were identified in both positive and negative ion modes, including 411 TGs, 84 diglycerides (DGs), 46 phosphatidylcholines (PCs), and 38 phosphatidylethanolamines (PEs), with these four types representing the highest quantities. The lipids were categorized into 21 sub‐classes, with 56.69% TG, 11.59% DG, 6.34% PC, 5.93% ceramide (Cer), 5.24% PE, 4.97% sphingomyelin (SM), and 1.93% phosphatidic acid (PA). As illustrated in Table 3, TGs, DGs, and PCs were the most prevalent lipid types across the three examined groups. Notably, the relative concentration of DGs in the SO group was significantly higher than that in both the PO and LO groups. Furthermore, the levels of sphingomyelin (Sph) and AcHex were significantly higher in the PO group compared to the other groups (*p* < 0.05).

**TABLE 3:** Lipid composition of experimental diets and donkey milk.

Lipid	Diet (%)			Donkey milk (%)			SEM	*p*
	SO	LO	PO	SO	LO	PO		
TG	68.5	69.04	67.92	91.55	91.98	91.36	0.396	0.189
DG	7.72	7.01	6.92	5.14^b^	4.42^a^	4.39^a^	0.056	0.013
SM	0.0248	0.0196	0.0233	0.81	1.01	1.06	0.03	0.473
Sph	0.1026	0.0714	0.0703	0.0839^a^	0.1148^a^	0.2096^b^	0.007	0.013
AcHex	6.18	5.52	4.20	0.0148^a^	0.0303^a^	0.0541^b^	0.002	0.001
PC	7.70	10.63	11.65	1.190	1.410	1.50	0.043	0.638
Cer	3.89	2.81	3.20	0.5209	0.2365	0.5332	0.149	0.196
PA	0.7087	0.6319	0.9515	0.2651	0.3187	0.2893	0.009	0.665
PE	0.245	0.3409	0.4697	0.1536	0.1778	0.1568	0.006	0.732
FA	0.0601	0.0754	0.0455	0.0998	0.1089	0.1866	0.01	0.378
HexCer	1.24	1.05	1.10	0.0634	0.0792	0.0929	0.003	0.386
MG	0.0583	0.0455	0.0479	0.0326	0.034	0.0514	0.002	0.268
PG	0.3061	0.2641	0.3681	0.0311	0.0274	0.0537	0.002	0.160
Co	0.0933	0.0938	0.0903	0.0209	0.0233	0.0265	0.001	0.487
MGMG	0.0113	0.0088	0.0086	0.0158	0.0176	0.0159	0.001	0.868
PS	0.0101	0.0146	0.0124	0.0098	0.0118	0.0095	0.000	0.574
CL	0.0189	0.0233	0.0232	0.0038	0.0034	0.007	0.000	0.123
PI	0.0697	0.0914	0.0679	0.0037	0.0043	0.0053	0.000	0.329
ChE	0.0062	0.0048	0.0068	0.0037	0.0016	0.0044	0.002	0.188
MGDG	0.32	0.27	0.27	0.0002	0.0003	0.0002	0.000	0.823

Abbreviations: AcHex, diacylatehexosyl; Cer, ceramides; CL, cardiolipin; Co, coenzyme; DG, diglyceride; HexCer, hexosylceramide; MG, monoglyceride; PA, phosphatidic acid; PC, phosphatidylcholine; PE, phosphatidylethanolamine; PG, phosphatidylglycerol; PI, phosphatidylinositol; PS, phosphatidylserine; SEM, standard error of mean; SM, sphingomyelin; Sph, sphingolipid; TG, triglyceride.

### 3.3 Differential Lipids and Metabolic Pathway

**FIGURE 2:**
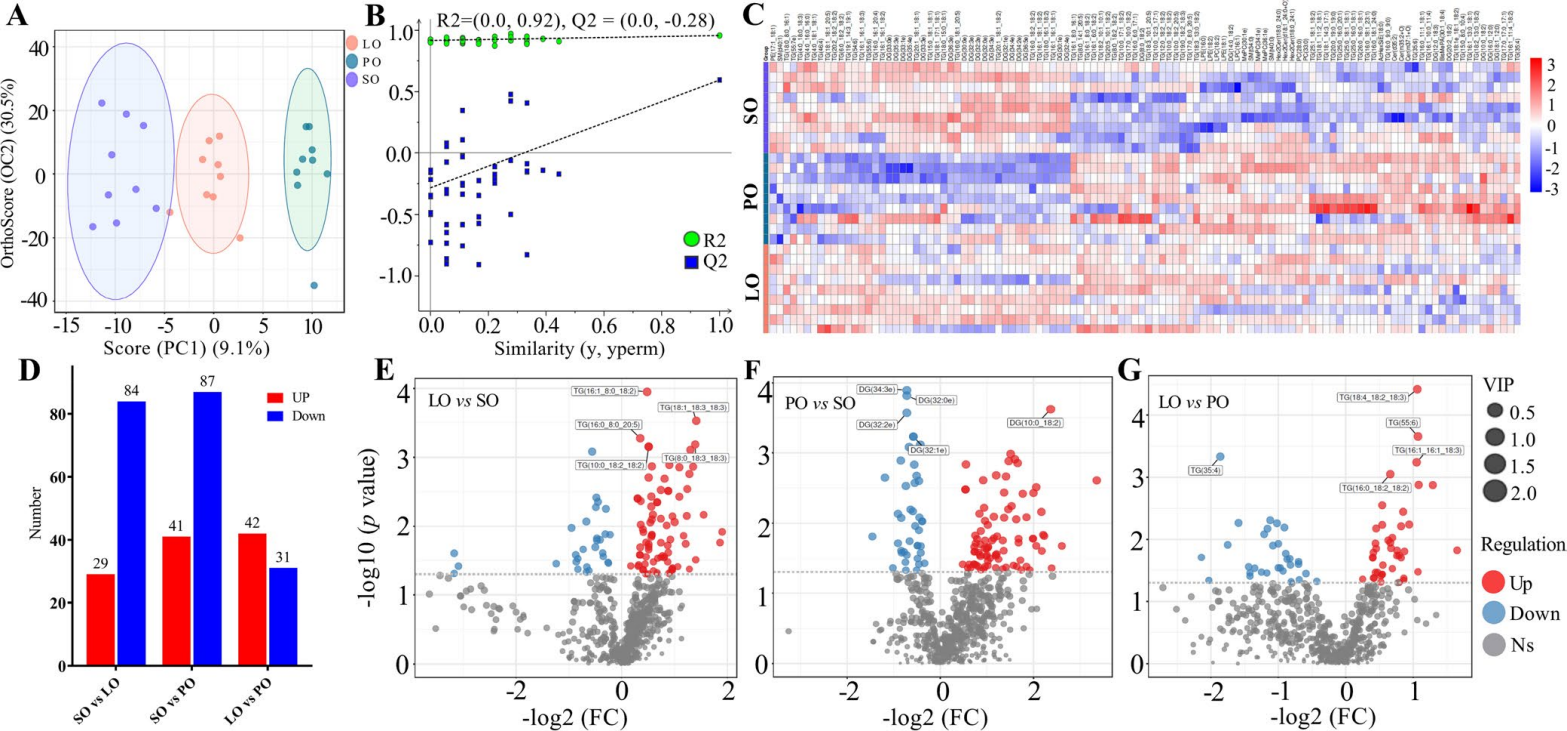
Multivariate analysis of lipidomics in donkey milk lipids. OPLS‐DA plot (A), corresponding OPLS‐DA validation plots (B), heatmap of hierarchical clustering analysis (red indicates increased, blue indicates decreased) (C), number of difference lipids (D), volcano diagrams of different donkey milk of lipids between the LO and PO group (E), the LO versus the SO group (F), the PO versus the SO group (G).

To enhance the evaluation of lipid variations among the three groups, we employed OPLS‐DA and heatmap analysis. As shown in Figure 2A, lipids in the three groups were clearly distinct. As observed in Figure 2B, *R* ^2^ = (0.0, 0.92) and *Q* ^2^ = (0.0, −0.28) suggest a robust model with no overfitting. In total, 110 differential lipids were identified, encompassing 64 TGs, 24 DGs, 2 PCs, 2 LPCs, 3 MePCs, 1 PE, 3 LPEs, 3 SMs, 3 Cers, 1 diacylatehexosyl (AcHex), 1 hexosylceramide (HexCer), and 1 BisMePA (Table S1). TG(18:2_13:0_18:2) was the potential marker for the discrimination between different donkey milk. A heatmap derived from hierarchical clustering analysis was constructed to illustrate these differences more effectively in Figure 2C. The analysis revealed that 29 upregulated and 84 downregulated lipids were identified for the SO versus LO comparison, 41 upregulated and 87 downregulated lipids were identified for the SO versus PO comparison, and 42 upregulated and 31 downregulated lipids were identified for the LO versus PO comparison, applying VIP > 1 and *p* < 0.05, as shown in Figure 2D. Volcano plots further illustrated the alterations in donkey milk lipids due to different dietary oils (Figure 2E–G). DGs were the main difference between the PO and SO groups.

**FIGURE 3:**
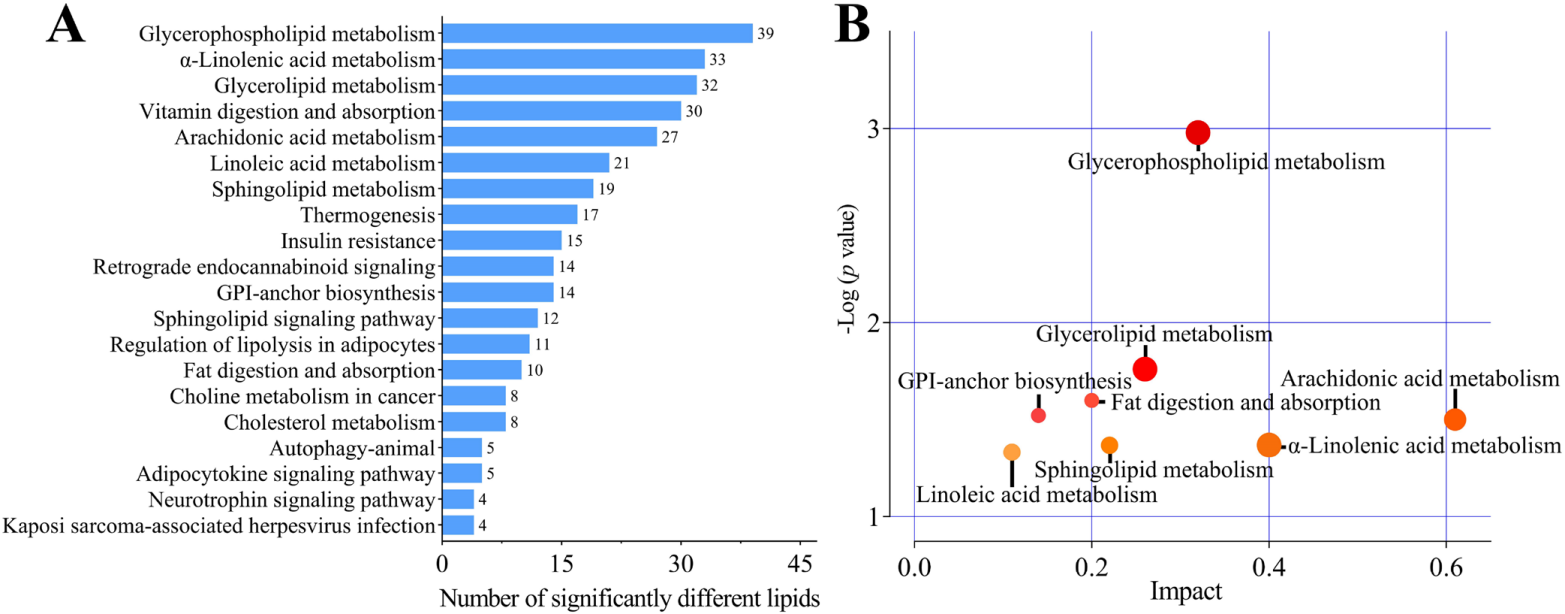
KEGG enrichment analysis. The number of significantly different lipids enriched in the KEGG metabolic pathway analysis (A) and the metabolic view map of the significant metabolic pathways of donkey milk lipids between SO, LO, and PO groups (B).

Analysis of the 110 differential lipids by KEGG pathway analysis revealed enrichment in 20 metabolic pathways. These included glycerophospholipid metabolism, glycerolipid metabolism, fat digestion and absorption, glycosylphosphatidylinositol (GPI)‐anchor biosynthesis, arachidonic acid metabolism, alpha‐linolenic acid metabolism, and sphingolipid metabolism (Figure 3A). The eight most significant metabolic pathways obtained included glycerophospholipid metabolism, glycerolipid metabolism, fat digestion and absorption, GPI‐anchor biosynthesis, arachidonic acid metabolism, alpha‐linolenic acid metabolism, sphingolipid metabolism, and linoleic acid metabolism (Figure 3B).

### 3.4 Voc Profiles of Donkey Milk

**FIGURE 4:**
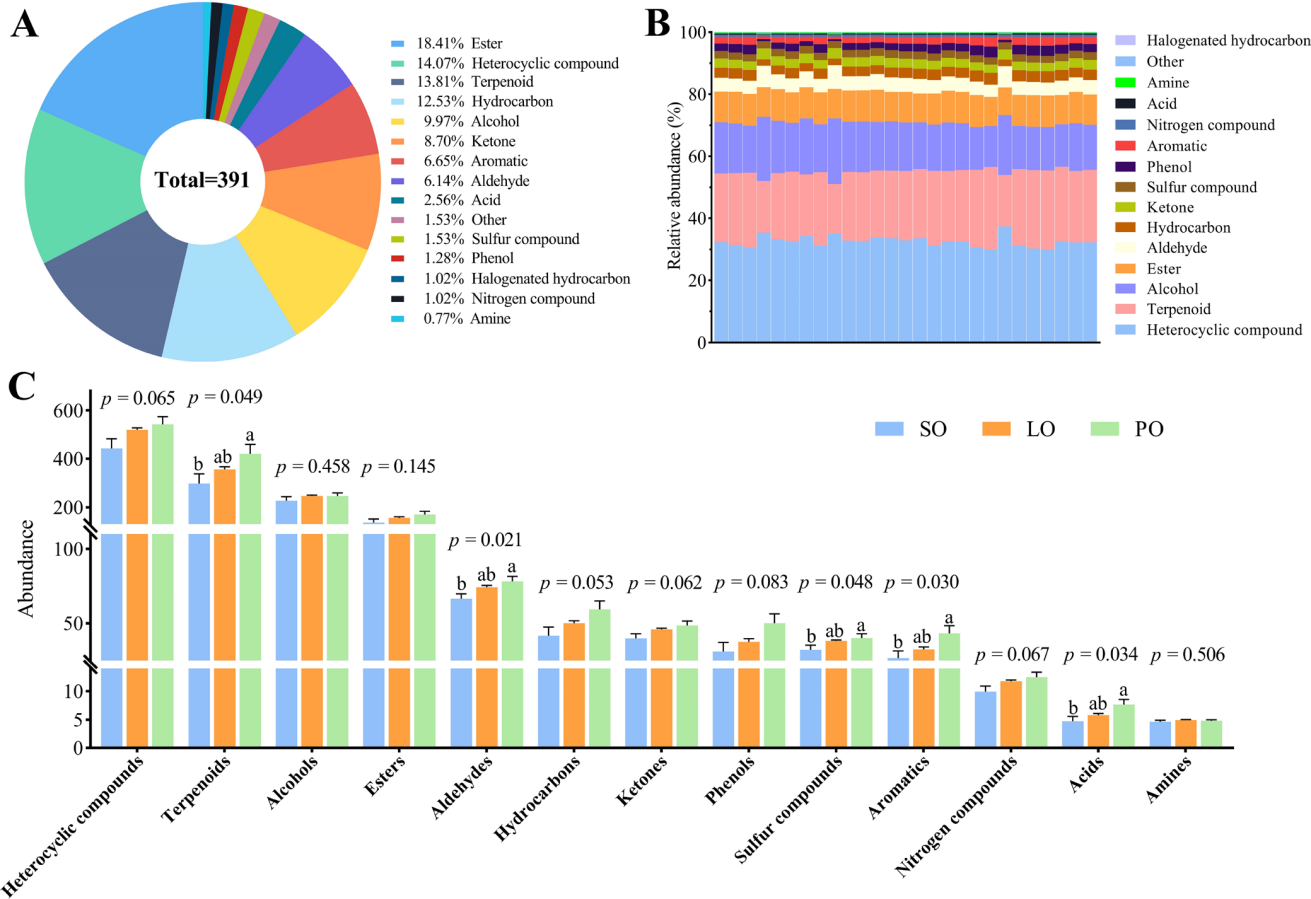
Qualitative analysis results for VOC compositions of different donkey milks. Quantity percentages of VOCs for SO, PO, and LO (A), content percentages of VOCs (the height of the rectangle indicates the percentage of each subclass in relation to the current item) (B), relative VOC contents (% total VOC composition) for SO, PO, and LO (C), values shown as mean ± SME (*n* = 9), where different letters indicate significant difference (*p* < 0.05).

A total of 391 VOCs were detected in donkey milk, comprising 72 esters, 55 heterocyclic compounds, 54 terpenoids, 49 hydrocarbons, 39 alcohols, and 34 ketones. Esters constituted the most significant proportion, followed by heterocyclic compounds, terpenoids, and hydrocarbons (Figure 4A). Heterocyclic compounds were predominant in terms of relative abundance within the donkey milk, as depicted in Figure 4B. The VOC levels for terpenoids, aldehydes, hydrocarbons, ketones, sulfur compounds, phenols, aromatics, and acids were notably higher in the PO group compared to those in the SO group (Figure 4C).

### 3.5 Differential Vocs in Donkey Milk

**FIGURE 5:**
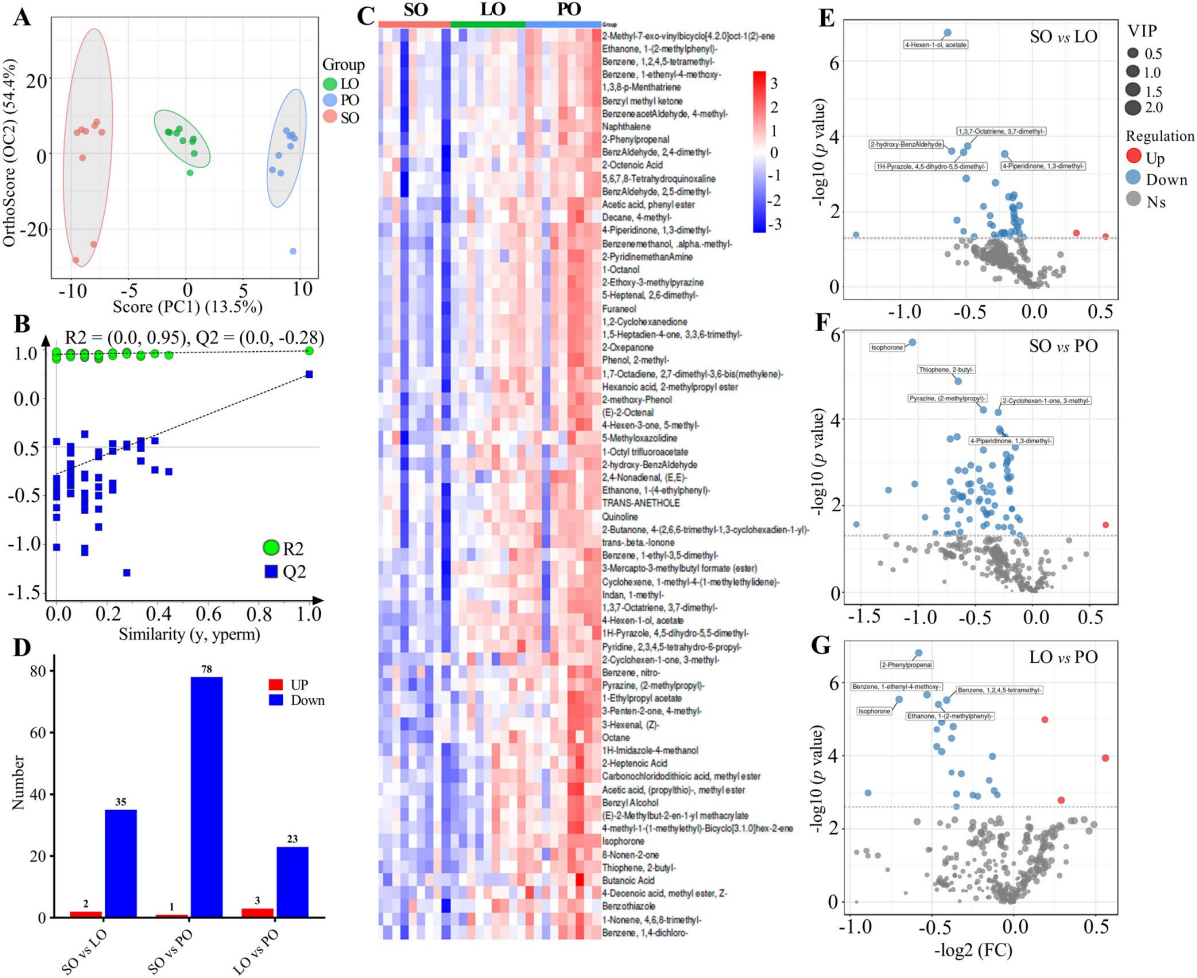
Difference analysis results for VOCs between the SO, PO, and LO groups. OPLS‐DA plot (A), corresponding OPLS‐DA validation plots (B), heatmap of hierarchical clustering analysis (red indicates increased, blue indicates decreased) (C), number of difference VOCs (D), volcano diagrams of different donkey milk of VOCs between the linseed oil (LO) and the palm oil (PO) group (E), the LO versus the soybean oil (SO) group (F), the PO versus the SO group (G).

To investigate the differences in VOCs comprehensively, we employed partial least squares regression to develop a model that correlates VOC expression with distinct groups of donkey milk, utilizing OPLS‐DA. Figure 5A distinctly illustrates the differentiation among milks from donkeys fed with SO, LO, and PO dietary supplements. The score scatter plot from the OPLS‐DA, with values R^2^ = (0.0, 0.95) and Q^2^ = (0.0, −0.28), indicates a well‐fitted model without overfitting (Figure 5B). Our analysis identified a total of 70 VOCs with significant differences, encompassing 13 heterocyclic compounds, 10 ketones, 9 esters, 9 aromatics, 8 aldehydes, 6 hydrocarbons, 5 alcohols, 4 terpenoids, 3 acids, 2 phenols, and 1 other compound, each demonstrating significant variance (VIP > 1 and *p* < 0.05, Table S2). The heatmap in Figure 5C distinctly showcases the pronounced difference in VOC content in the PO group compared to the SO group. Employing criteria of VIP > 1 and *p* < 0.05 for distinguishing diverse VOCs, we found 2 upregulated and 35 downregulated VOCs in the SO group compared to the LO group; 1 upregulated and 78 downregulated VOCs in the SO group compared to the PO group; and 3 upregulated and 23 downregulated VOCs in the LO group compared to the PO group (Figure 5D). The volcanic plots, from Figure 5E–G, offer a more intuitive representation of VOC variations among these groups.

## 4 Discussion

Donkey milk is easily absorbed and utilized by the body, offering essential FAs that stimulate appetite, demonstrate inflammatory and anti‐histamine effects, and provide benefits for improving allergies and problematic skin (Carminati and Tidona 2017). Compared to previous research on Dezhou donkey milk, which showed a milk fat content of 0.78%, the current study found significantly lower milk fat content in the SO (0.25%), LO (0.07%), and PO (0.26%) groups (Yu 2020). This observation is consistent with earlier studies indicating that the inclusion of plant and animal oils in the diets of dairy animals can lead to reduced milk fat content, a condition referred to as low milk fat syndrome in dairy production (Han et al. 2023). The protein (1.20%–1.80%), lactose (5.0%–8.0%), solids (9.0%–9.50%), and lactoferrin (9.0%–10.0%) levels in the milk are in agreement with previous studies (Guo et al. 2007; Yu 2020). Overall, the PO group exhibited a higher milk fat content compared to the SO and LO groups, although all groups presented with lower milk fat content than traditionally observed.

The study employed traditional LC–MS technology, the predominant method for lipid detection in milk, facilitating a more comprehensive identification of lipids in donkey milk. Previous research identified 72 TGs in both donkey milk and human milk (Chiofalo et al. 2011), and six lipid sub‐classes in donkey milk (Contarini et al. 2017). Furthermore, 335 lipid molecules were determined across different lactation stages using LC–MS (Li et al. 2020). Notably, this study identified 21 sub‐classes comprising 725 lipids in the SO, LO, and PO groups via LC–MS. Employing lipidomic approaches, additional compounds such as wax esters, zymosterol ester, cholesterol ester, AcHex, and derivatized lipids (BisMePA and dMePE) were detected, surpassing previous findings (Chiofalo et al. 2011; Contarini et al. 2017; Dugo et al. 2005; Li et al. 2020). Moreover, the study accurately identified the number of lipid isomers and double bonds, distinguishing the structure and position of specific lipids, exemplified by TG (10:0_10:1_10:1) and TG (10:0_10:1_12:4). In summary, novel methodologies have yielded a more detailed and expansive understanding of the lipid composition in donkey milk.

In this study, 110 differential lipids spanning nine sub‐classes were identified, with TG accounting for 58% and unsaturated fatty acids (UFAs) making up 88.2%. These findings align with prior research indicating that various dietary oils can modify the FA composition of milk, with TGs predominantly identified as the principal differing lipids in donkey milk (Bougouin et al. 2019; Li et al. 2020; Ren et al. 2023). The levels of TG (18:1_18:3_18:3), TG (8:0_18:3_18:3), TG (18:4_18:2_18:3), and TG (16:1_16:1_18:3) were higher for the discrimination of donkey milk in the LO group. Previous studies have demonstrated that while SFAs primarily serve as an energy source, they may also contribute to increased cholesterol and neutral fat levels within the body (Arne Astrup et al. 2020). Conversely, UFAs are noted for their significant roles in reducing cholesterol and inflammation, enhancing brain health, and mitigating the risk of heart disease (Cussotto et al. 2022; Murru et al. 2022; Thesing et al. 2020). The analysis indicated a notably higher concentration of DG in the SO group compared to the PO and LO groups. DG, a minor constituent of natural plant oils, is recognized for its potential to reduce visceral fat, curb weight gain, and decrease blood lipid levels by impeding fat accumulation (Hara et al. 1993; Murata et al. 1994). The levels of Sph were significantly higher in the SO and LO groups than in the PO group. SPs are an important component of the lipid bilayer in the milk fat globule membrane, closely related to brain development (Eckhardt et al. 2002). The KEGG pathways analysis of these lipids revealed that glycerophospholipid metabolism was the most implicated pathway in the observed lipid variations, followed by glycerolipid metabolism, GPI‐anchor biosynthesis, arachidonic acid metabolism, alpha‐linolenic acid metabolism, and linoleic acid metabolism, which are in agreement with previous studies (Li et al. 2020; Ren et al. 2023). However, this study uniquely identified significant involvement of sphingolipid metabolism and fat digestion and absorption pathways in the lipid variations. The identified significantly different lipids and related metabolic pathways may serve as potential biomarkers. In summary, the detection of a broader spectrum of lipids in donkey milk suggests potential modifications attributable to the dietary oil supplementation in donkey feed.

Aroma, taste, and appearance a serve as crucial metrics for assessing the quality of food, with VOCs playing a pivotal role in influencing food flavor. GC–MS analysis, renowned for its efficient separation capabilities and precise quantification with minimal sample requirements, is increasingly utilized for the analysis of VOCs (Papadimitropoulos et al. 2018). Previous research has identified 48 VOCs in van herby Cheese using raw and pasteurized milks from various species through GC–MS (Ocak et al. 2015), and 53 VOCs in goat milk cake (Tian et al. 2020). Additionally, an analysis of donkey milk powder employing both distillation extraction and solid‐phase microextraction methods revealed 54 VOCs via GC–MS (Xi et al. 2016), while 86 VOCs were identified in fermented milk following fermentation (Dan et al. 2019). In the current study, an advanced GC–MS technique facilitated the detection of 391 VOCs in the milk of donkeys fed with SO, LO, and PO. The detected VOCs encompassed a diverse range of chemical families, including esters, heterocyclic compounds, terpenoids, hydrocarbons, alcohols, ketones, aromatic compounds, aldehydes, acids, and others, along with sulfur compounds, phenols, halogenated hydrocarbons, nitrogen compounds, and amines. This finding aligns with prior research, which identified ketones, aldehydes, acids, alcohols, and esters as fundamental VOCs in milk (Imhof and Bosset 1994; Krings and Berger 1998). The detection of a higher quantity of VOCs in this study not only signifies an enhancement in analytical technology but also provides a more comprehensive elucidation of the VOC composition in donkey milk. Intriguingly, compared to a previous study that identified 45 VOCs (Ren et al. 2023), the significant increase in the number of VOCs detected in this study could be attributed to the inclusion of dietary oils in the donkeys' diet.

Previous research has established that dietary flavors are transferred to milk, a process that encompasses both direct transmission and biological transformation (Debong and Loos 2020; Mennella and Beauchamp 1991; Spahn et al. 2019). This study investigated the impact of various oils used to feed donkeys on the flavor profile of the resulting donkey milk. Utilizing an untargeted metabolomics approach, the study aimed to identify and evaluate changes in the overall composition of VOCs in milk from donkeys fed different dietary oils. Significant differences were observed in the concentrations of terpenoids, aldehydes, hydrocarbons, ketones, sulfur compounds, phenols, aromatics, and acids, with higher levels found in the group fed PO compared to those fed SO and LO.

Terpenoids, a predominant class of secondary plant metabolites commonly found in plant‐based diets, are known for their natural scents and have been linked to lipid oxidation (Kiralan and Ramadan 2016; Poulopoulou et al. 2012). Previous studies have shown that terpenes can be transferred to milk when sheep are orally administered these compounds indoors (Poulopoulou et al. 2012), suggesting that feeding PO to donkeys could increase the terpenoid content in their milk. Furthermore, ketones and aldehydes, which are produced from PUFAs in vegetable oils, were found to contribute to the unique flavor profiles of milk. These compounds, generated during the FA β‐oxidation process, are characterized by their creamy, fruity, floral, and moldy flavors and are significantly influenced by the FA composition of fats and oils (Cao et al. 2014; Christie and Harwood 2020). The study identified specific compounds, including ethanol, acetone, propanol, butanal, 2‐pentenal, 2‐hexenal, 2,4‐heptadienal, and 2,4‐nonadienal, as strongly correlated with the initial content of α‐linolenic acid. Additionally, octanal, octanone, nonanal, nonanone, decanal, decanone, and 2‐decanal were recognized as key markers associated with the initial content of oleic acid (Cao et al. 2014), indicating that the composition of aldehydes, ketones, phenols, aromatics, and acids in milk is affected by the FA composition of different lipids. The study also found that 70 VOCs exhibited significant differences, with 68 of these compounds showing higher concentrations in the PO group compared to the SO and LO groups. Notably, 2‐hydroxy‐benzaldehyde was more prevalent in the PO group, while the content of quinoline was similar in both the LO and PO groups. This discovery is crucial for understanding the variations in FA composition across different oil types and their influence on milk lipids, which ultimately affects the flavor profile of milk. Overall, this research highlights the significant impact of consuming various dietary oils on the VOC composition of donkey milk.

## 5 Conclusions

The comprehensive lipidomics and flavoromics analysis of donkeys fed with different oils has mainly found that dietary enrichment of UFA oil largely affects the composition of lipid molecules in donkey milk. The 110 differential lipids out of 725 lipids were detected, which were most relevant to the eight metabolic pathways, including glycerophospholipid and glycerolipid metabolism. The 70 differential VOCs out of 391 VOCs were detected in donkey milk, mainly heterocyclic compounds. Terpenoids, alcohols, aldehydes, sulfur compounds, aromatics, and acids were significantly more abundant in the palm oil group compared to the other groups. This finding reveals the potential of dietary oils to improve the lipid composition and VOC profiles of donkey milk.

## Author Contributions

**Wei Ren:** conceptualization (equal), data curation (equal), investigation (equal), methodology (equal), software (equal), writing – original draft (lead), writing – review and editing (equal). **Lingyun Sun:** data curation (equal), formal analysis (equal), funding acquisition (equal), resources (equal). **Xinyi Du:** formal analysis (equal), investigation (equal), methodology (equal), validation (equal). **Yile Chen:** data curation (equal), methodology (equal), resources (equal), software (equal). **Yinghui Chen:** resources (equal), software (equal), visualization (equal). **Huili Liang:** data curation (equal), formal analysis (equal), methodology (equal), resources (equal), validation (equal), visualization (equal). **Xiyan Kou:** formal analysis (equal), resources (equal), software (equal). **Muhammad Zahoor Khan:** conceptualization (equal), supervision (equal), validation (equal), visualization (equal). **Changfa Wang:** conceptualization (equal), funding acquisition (equal), investigation (equal), project administration (equal), supervision (equal), validation (equal), visualization (equal). **Mengmeng Li:** conceptualization (equal), funding acquisition (equal), methodology (equal), project administration (equal), supervision (equal), validation (equal).

## Ethics Statement

All animal experiments received approval from the Animal Care and Use Committee of Liaocheng University (2023022706).

## Consent

The authors have nothing to report.

## Conflicts of Interest

The authors declare no conflicts of interest.

## Supporting information


Tables S1–S2.

## Data Availability

The datasets used during the current study are available from the corresponding author on reasonable request.
