# Supplementary material for: Dietary Oils Alter Lipid and Volatile Compound Profiles of Donkey Milk: A Comprehensive Analysis
Source: Food Sci Nutr. 2025 May 20;13(5):e70291. doi: 10.1002/fsn3.70291 (PMC12121528; doi:10.1002/fsn3.70291)
Supplement: Supplementary file 1 — Tables S1–S2. [file FSN3-13-e70291-s001.docx]

**Supplementary Tables**

**Table S1** Differential lipids in SO, PO, and LO groups

| No. | Lipid | Class | Category | MainIon | mz | Rt (min) | VIP | *p* value |
| --- | --- | --- | --- | --- | --- | --- | --- | --- |
| 1 | AcHexSiE(18:0) | AcHex | SL | M+NH4 | 860.7338 | 19.839 | 1.9188 | 0.0053 |
| 2 | Cer(d35:2) | Cer | SP | M+H | 550.5194 | 10.627 | 1.8490 | 0.0255 |
| 3 | Cer(m35:2+O) | Cer | SP | M+H | 550.5194 | 10.629 | 1.8758 | 0.0221 |
| 4 | Cer(m37:1+O) | Cer | SP | M+H | 580.5663 | 15.779 | 1.5149 | 0.0243 |
| 5 | Hex2Cer(d18:1_24:0+O) | HexCer | SP | M+H | 990.7451 | 16.452 | 1.6517 | 0.0469 |
| 6 | Hex2Cer(t18:0_24:0) | HexCer | SP | M+H | 992.7608 | 17.629 | 1.7142 | 0.0139 |
| 7 | Hex2Cer(t18:0_24:1) | HexCer | SP | M+H | 990.7451 | 16.450 | 1.6522 | 0.0465 |
| 8 | SM(d40:1) | SM | SP | M+H | 787.6688 | 16.410 | 1.7201 | 0.0340 |
| 9 | SM(t34:0) | SM | SP | M+H | 721.5854 | 12.343 | 2.3520 | 0.0009 |
| 10 | SM(t40:0) | SM | SP | M+HCOO | 849.6702 | 16.673 | 1.7789 | 0.0437 |
| 11 | PC(28:0) | PC | GP | M+H | 678.5068 | 11.495 | 1.7387 | 0.0232 |
| 12 | PC(33:0) | PC | GP | M+H | 748.5851 | 15.212 | 1.3579 | 0.0345 |
| 13 | LPC(16:1) | PC | GP | M+H | 494.3241 | 2.242 | 1.8732 | 0.0323 |
| 14 | LPC(18:2) | PC | GP | M+HCOO | 564.3307 | 2.492 | 2.2520 | 0.0059 |
| 15 | MePC(30:1e) | PC | GP | M+NH4 | 721.5854 | 12.345 | 2.3284 | 0.0015 |
| 16 | MePC(34:1e) | PC | GP | M+NH4 | 777.6480 | 15.347 | 1.9411 | 0.0187 |
| 17 | MePC(36:1e) | PC | GP | M+NH4 | 805.6793 | 16.666 | 1.9866 | 0.0141 |
| 18 | LPE(16:0) | PE | GP | M-H | 452.2783 | 3.347 | 2.2217 | 0.0020 |
| 19 | LPE(18:1) | PE | GP | M+H | 480.3085 | 3.902 | 2.2143 | 0.0127 |
| 20 | LPE(18:2) | PE | GP | M+H | 478.2928 | 2.620 | 2.1651 | 0.0054 |
| 21 | PE(17:1_18:1) | PE | GP | M-H | 728.5236 | 14.279 | 1.2112 | 0.0422 |
| 22 | BisMePA(30:1_18:4) | PA | GP | M+Na | 913.6657 | 19.209 | 1.1555 | 0.0257 |
| 23 | DG(10:0_18:2) | DG | GL | M+NH4 | 526.4466 | 11.313 | 2.5167 | 0.0001 |
| 24 | DG(12:0_18:3) | DG | GL | M+NH4 | 552.4623 | 11.725 | 1.4741 | 0.0436 |
| 25 | DG(14:0_18:2) | DG | GL | M+NH4 | 582.5092 | 14.313 | 2.2794 | 0.0016 |
| 26 | DG(16:0_12:0) | DG | GL | M+Na | 535.4333 | 14.027 | 2.0319 | 0.0076 |
| 27 | DG(18:1_12:0) | DG | GL | M+Na | 561.4489 | 14.053 | 2.0161 | 0.0072 |
| 28 | DG(20:2_18:2) | DG | GL | M+NH4 | 662.5718 | 15.861 | 1.5071 | 0.0345 |
| 29 | DG(30:0e) | DG | GL | M+Na | 549.4853 | 20.861 | 1.7960 | 0.0155 |
| 30 | DG(30:1e) | DG | GL | M+Na | 547.4697 | 20.453 | 1.5286 | 0.0351 |
| 31 | DG(32:0e) | DG | GL | M+Na | 577.5166 | 21.239 | 2.3363 | 0.0007 |
| 32 | DG(32:1e) | DG | GL | M+Na | 575.5010 | 20.898 | 2.0961 | 0.0027 |
| 33 | DG(32:2e) | DG | GL | M+H | 551.5034 | 21.269 | 2.3152 | 0.0002 |
| 34 | DG(32:3e) | DG | GL | M+H | 549.4877 | 20.852 | 1.7179 | 0.0245 |
| 35 | DG(32:4e) | DG | GL | M+H | 547.4721 | 20.453 | 1.5286 | 0.0351 |
| 36 | DG(33:0e) | DG | GL | M+Na | 591.5323 | 21.411 | 1.7342 | 0.0318 |
| 37 | DG(33:1e) | DG | GL | M+Na | 589.5166 | 21.031 | 2.0751 | 0.0021 |
| 38 | DG(33:3e) | DG | GL | M+H | 563.5034 | 21.051 | 1.6567 | 0.0407 |
| 39 | DG(34:2e) | DG | GL | M+Na | 601.5166 | 20.867 | 1.7033 | 0.0259 |
| 40 | DG(34:3e) | DG | GL | M+H | 577.5190 | 21.244 | 2.3515 | 0.0006 |
| 41 | DG(34:4e) | DG | GL | M+H | 575.5034 | 20.883 | 2.0961 | 0.0027 |
| 42 | DG(35:3e) | DG | GL | M+H | 591.5347 | 21.414 | 1.7458 | 0.0336 |
| 43 | DG(35:4e) | DG | GL | M+H | 589.5190 | 21.035 | 2.0513 | 0.0030 |
| 44 | DG(36:3e) | DG | GL | M+H | 605.5503 | 21.584 | 2.1144 | 0.0019 |
| 45 | DG(36:5e) | DG | GL | M+H | 601.5190 | 20.873 | 1.7033 | 0.0259 |
| 46 | DG(8:0_18:2) | DG | GL | M+NH4 | 498.4153 | 9.842 | 1.9429 | 0.0062 |
| 47 | TG(10:0_12:3_17:1) | TG | GL | M+H | 673.5402 | 17.402 | 1.8817 | 0.0111 |
| 48 | TG(10:0_17:1_18:2) | TG | GL | M+NH4 | 776.6763 | 19.132 | 2.1721 | 0.0013 |
| 49 | TG(10:0_18:2_18:2) | TG | GL | M+Na | 793.6317 | 18.903 | 1.9869 | 0.0008 |
| 50 | TG(10:0_18:2_20:5) | TG | GL | M+H | 793.6341 | 18.926 | 1.9869 | 0.0008 |
| 51 | TG(12:0_18:2_18:2) | TG | GL | M+Na | 821.6630 | 19.507 | 2.0717 | 0.0008 |
| 52 | TG(15:0_18:1_18:1) | TG | GL | M+NH4 | 862.7858 | 21.042 | 1.7221 | 0.0126 |
| 53 | TG(15:0_8:0_10:4) | TG | GL | M+NH4 | 606.4728 | 13.502 | 1.7289 | 0.0371 |
| 54 | TG(16:0_11:1_18:1) | TG | GL | M+NH4 | 778.6919 | 19.934 | 1.1337 | 0.0359 |
| 55 | TG(16:0_16:1_20:4) | TG | GL | M+NH4 | 870.7545 | 20.138 | 1.3360 | 0.0190 |
| 56 | TG(16:0_18:1_20:5) | TG | GL | M+H | 879.7436 | 21.469 | 1.8930 | 0.0082 |
| 57 | TG(16:0_18:1_23:1) | TG | GL | M+NH4 | 946.8797 | 22.009 | 1.6595 | 0.0095 |
| 58 | TG(16:0_18:1_24:0) | TG | GL | M+NH4 | 962.9110 | 22.438 | 1.3927 | 0.0486 |
| 59 | TG(16:0_18:2_18:2) | TG | GL | M+NH4 | 872.7702 | 20.028 | 2.2126 | 0.0011 |
| 60 | TG(16:0_6:0_17:1) | TG | GL | M+NH4 | 696.6137 | 18.615 | 1.5894 | 0.0026 |
| 61 | TG(16:0_9:0_9:0) | TG | GL | M+NH4 | 628.5511 | 13.222 | 1.6209 | 0.0369 |
| 62 | TG(16:1_10:0_11:4) | TG | GL | M+NH4 | 660.5198 | 14.871 | 1.4751 | 0.0272 |
| 63 | TG(16:1_11:4_18:2) | TG | GL | M+NH4 | 768.6137 | 17.524 | 1.6390 | 0.0437 |
| 64 | TG(16:1_13:0_18:1) | TG | GL | M+NH4 | 806.7232 | 20.179 | 1.9590 | 0.0150 |
| 65 | TG(16:1_16:1_18:1) | TG | GL | M+NH4 | 846.7545 | 20.458 | 1.6448 | 0.0201 |
| 66 | TG(16:1_16:1_18:2) | TG | GL | M+NH4 | 844.7389 | 20.033 | 1.2027 | 0.0291 |
| 67 | TG(16:1_16:1_18:3) | TG | GL | M+Na | 847.6786 | 19.611 | 1.0112 | 0.0005 |
| 68 | TG(16:1_18:1_18:2) | TG | GL | M+NH4 | 872.7702 | 20.505 | 1.8693 | 0.0030 |
| 69 | TG(16:1_6:0_18:2) | TG | GL | M+NH4 | 706.5980 | 17.230 | 1.4042 | 0.0087 |
| 70 | TG(16:1_8:0_16:1) | TG | GL | M+NH4 | 708.6137 | 18.007 | 1.9202 | 0.0089 |
| 71 | TG(16:1_8:0_18:2) | TG | GL | M+Na | 739.5847 | 18.071 | 1.0237 | 0.0008 |
| 72 | TG(17:0_10:0_18:2) | TG | GL | M+NH4 | 778.6919 | 19.675 | 1.5620 | 0.0466 |
| 73 | TG(17:0_17:0_17:1) | TG | GL | M+Na | 869.7569 | 20.624 | 1.5582 | 0.0241 |
| 74 | TG(17:0_8:0_20:5) | TG | GL | M+H | 755.6184 | 19.103 | 1.1064 | 0.0449 |
| 75 | TG(18:0_15:0_18:1) | TG | GL | M+NH4 | 864.8015 | 21.049 | 1.6345 | 0.0335 |
| 76 | TG(18:0_16:1_18:2) | TG | GL | M+NH4 | 874.7858 | 20.503 | 1.9516 | 0.0007 |
| 77 | TG(18:0_8:0_16:1) | TG | GL | M+NH4 | 738.6606 | 18.752 | 1.6776 | 0.0169 |
| 78 | TG(18:1_11:2_18:1) | TG | GL | M+H | 785.6654 | 20.204 | 1.2814 | 0.0267 |
| 79 | TG(18:1_14:3_17:1) | TG | GL | M+H | 811.6810 | 20.244 | 1.7135 | 0.0216 |
| 80 | TG(18:1_17:1_18:1) | TG | GL | M+NH4 | 888.8015 | 21.025 | 1.6727 | 0.0304 |
| 81 | TG(18:1_18:1_20:5) | TG | GL | M+NH4 | 922.7858 | 19.659 | 1.6181 | 0.0005 |
| 82 | TG(18:1e_18:1_18:2) | TG | GL | M+NH4 | 886.8222 | 21.369 | 1.4498 | 0.0026 |
| 83 | TG(18:2_10:1_10:1) | TG | GL | M+NH4 | 676.5511 | 15.056 | 1.3081 | 0.0274 |
| 84 | TG(18:2_13:0_18:2) | TG | GL | M+NH4 | 830.7232 | 19.824 | 2.2882 | 0.0004 |
| 85 | TG(18:3_10:1_20:5) | TG | GL | M+H | 789.6028 | 17.624 | 1.3602 | 0.0118 |
| 86 | TG(18:3_13:0_18:2) | TG | GL | M+NH4 | 828.7076 | 19.241 | 1.7537 | 0.0219 |
| 87 | TG(19:0_18:2_18:3) | TG | GL | M+NH4 | 912.8015 | 20.256 | 1.1431 | 0.0032 |
| 88 | TG(19:1_14:3_19:1) | TG | GL | M+NH4 | 870.7545 | 19.608 | 1.4779 | 0.0194 |
| 89 | TG(20:0_19:0_19:0) | TG | GL | M+H | 947.9001 | 22.050 | 1.7115 | 0.0121 |
| 90 | TG(20:1_18:1_18:1) | TG | GL | M+NH4 | 930.8484 | 21.537 | 1.8987 | 0.0064 |
| 91 | TG(20:1_18:1_18:2) | TG | GL | M+NH4 | 928.8328 | 21.198 | 2.3139 | 0.0007 |
| 92 | TG(20:2_18:2_18:2) | TG | GL | M+NH4 | 924.8015 | 20.536 | 1.1682 | 0.0190 |
| 93 | TG(20:5_10:1_18:2) | TG | GL | M+H | 791.6184 | 18.288 | 1.2485 | 0.0125 |
| 94 | TG(25:0_16:0_18:1) | TG | GL | M+NH4 | 976.9267 | 22.540 | 1.5661 | 0.0336 |
| 95 | TG(25:0_16:0_20:1) | TG | GL | M+NH4 | 1004.9580 | 22.768 | 1.8805 | 0.0046 |
| 96 | TG(25:0_18:1_18:1) | TG | GL | M+NH4 | 1002.9423 | 22.530 | 1.7362 | 0.0226 |
| 97 | TG(25:1_18:1_18:2) | TG | GL | M+NH4 | 998.9110 | 21.962 | 1.7563 | 0.0172 |
| 98 | TG(26:0_16:0_18:1) | TG | GL | M+NH4 | 990.9423 | 22.669 | 1.7466 | 0.0204 |
| 99 | TG(35:4) | TG | GL | M+H | 617.4776 | 14.996 | 1.6706 | 0.0021 |
| 100 | TG(35:6) | TG | GL | M+NH4 | 630.4728 | 12.335 | 1.8596 | 0.0050 |
| 101 | TG(46:4) | TG | GL | M+NH4 | 788.6763 | 18.176 | 1.7274 | 0.0045 |
| 102 | TG(4:0_16:0_16:0) | TG | GL | M+NH4 | 656.5824 | 18.212 | 1.8086 | 0.0189 |
| 103 | TG(4:0_18:0_18:3) | TG | GL | M+NH4 | 706.5980 | 16.520 | 1.8389 | 0.0369 |
| 104 | TG(4:0_18:1_18:1) | TG | GL | M+NH4 | 708.6137 | 17.389 | 1.9243 | 0.0039 |
| 105 | TG(54:6) | TG | GL | M+NH4 | 896.7702 | 19.660 | 1.2424 | 0.0071 |
| 106 | TG(55:6) | TG | GL | M+NH4 | 910.7858 | 19.607 | 1.1407 | 0.0001 |
| 107 | TG(55:7e) | TG | GL | M+Na | 899.7463 | 21.612 | 1.4112 | 0.0170 |
| 108 | TG(6:0_18:2_18:2) | TG | GL | M+NH4 | 732.6137 | 16.413 | 1.2651 | 0.0252 |
| 109 | TG(8:0_14:1_20:5) | TG | GL | M+H | 711.5558 | 17.229 | 1.3853 | 0.0166 |
| 110 | TG(8:0_18:2_18:2) | TG | GL | M+Na | 765.6004 | 18.168 | 1.1382 | 0.0048 |

Abbreviations: mz, mass-to-chargeratio; VIP, variable importance in projection; RT: retention time; TG, triglyceride; DG, diglyceride; PC, phosphatidylcholine; MePC, methyl phosphatidylcholine; LPC, lysophosphatidylcholine; PE, phosphatidylethanolamine; LPE, lysophosphatidylethanolamine; Cer, ceramide; Hex2Cer, simple glc series; SM, sphingomyelin; BisMePA, bis-methyl phosphatidic acid; AcHexSiE, acylGlcSitosterol ester; GL, glycerolipid; GP, glycerophospholipid; SP, sphingolipid; SL, sterol lipid

**Table S2** Formation of volatile organic compounds in donkey milk for different oils

| No. | Compounds (×10^5^) | NIST_RI | CAS | SO | LO | PO | SEM | VIP | *P* value |
| --- | --- | --- | --- | --- | --- | --- | --- | --- | --- |
| 1 | (E)-2-Methylbut-2-en-1-yl Methacrylate | 1088 | 88142-95-4 | 2.9488 | 3.2276 | 3.7495 | 0.107 | 1.6461 | 0.0039 |
| 2 | (E)-2-Octenal | 1060 | 2548-87-0 | 1.9642 | 2.1394 | 2.2177 | 0.036 | 1.5070 | 0.0101 |
| 3 | 1,2-Cyclohexanedione | 1062 | 765-87-7 | 8.9667 | 9.9799 | 10.5327 | 0.189 | 1.8062 | 0.0008 |
| 4 | 3,7-Dimethyl-1,3,7-Octatriene | 1048 | 502-99-8 | 0.3841 | 0.5386 | 0.6052 | 0.025 | 1.9162 | 0.0001 |
| 5 | 1,3,8-p-Menthatriene | 1119 | 18368-95-1 | 2.3068 | 2.6594 | 3.6090 | 0.168 | 1.6285 | 0.0042 |
| 6 | 3,3,6-Trimethyl-1,5-Heptadien-4-One | 1062 | 546-49-6 | 30.9787 | 34.4865 | 36.3334 | 0.622 | 1.8849 | 0.0003 |
| 7 | 2,7-Dimethyl-3,6-Bis (Methylene)-1,7-Octadiene | 1083 | 16714-60-6 | 0.4578 | 0.5383 | 0.5989 | 0.021 | 1.4157 | 0.0256 |
| 8 | 1-Ethylpropyl Acetate | 793 | 620-11-1 | 31.8821 | 33.3920 | 36.2776 | 0.549 | 1.7786 | 0.0014 |
| 9 | 4,6,8-Trimethyl-1-Nonene | 1012 | 54410-98-9 | 0.1413 | 0.1650 | 0.1963 | 0.007 | 1.5943 | 0.0045 |
| 10 | 1-Octanol | 1072 | 111-87-5 | 4.2508 | 4.6770 | 4.8891 | 0.083 | 1.6709 | 0.0029 |
| 11 | 1-Octyl Trifluoroacetate | 1059 | 2561-21-9 | 0.6093 | 0.6829 | 0.6929 | 0.015 | 1.2486 | 0.0359 |
| 12 | 1H-Imidazole-4-Methanol | 1080 | 822-55-9 | 0.5565 | 0.6590 | 0.7477 | 0.024 | 1.7860 | 0.0015 |
| 13 | 4,5-Dihydro-5,5-Dimethyl-1H-Pyrazole | 983 | 4320-85-8 | 0.0955 | 0.1365 | 0.1496 | 0.007 | 1.7315 | 0.0004 |
| 14 | (E, E)-2,4-Nonadienal | 1216 | 5910-87-2 | 0.0797 | 0.1029 | 0.1907 | 0.014 | 1.5327 | 0.0073 |
| 15 | 4-(2,6,6-Trimethyl-1,3-Cyclohexadien-1-Yl)-2-Butanone | 1424 | 20483-36-7 | 0.1321 | 0.1913 | 0.2529 | 0.016 | 1.4421 | 0.0155 |
| 16 | 3-Methyl-2-Cyclohexen-1-One | 1075 | 1193-18-6 | 2.2226 | 2.4899 | 2.7286 | 0.069 | 1.6146 | 0.0005 |
| 17 | 2-Ethoxy-3-Methylpyrazine | 1065 | 32737-14-7 | 15.6574 | 17.2645 | 18.1991 | 0.307 | 1.8027 | 0.0009 |
| 18 | 2-Heptenoic Acid | 1081 | 18999-28-5 | 1.3439 | 1.4548 | 1.7314 | 0.057 | 1.5366 | 0.0088 |
| 19 | 2-Methyl-7-Exo-Vinylbicyclo [4.2.0] Oct-1(2)-Ene | 1112 | 107914-89-6 | 0.1088 | 0.1168 | 0.1579 | 0.007 | 1.5228 | 0.0067 |
| 20 | 2-Octenoic Acid | 1181 | 1470-50-4 | 0.5104 | 0.6125 | 0.6737 | 0.022 | 1.5581 | 0.0272 |
| 21 | 2-Oxepanone | 1065 | 502-44-3 | 7.6637 | 8.3739 | 8.8197 | 0.153 | 1.6705 | 0.0041 |
| 22 | 2-Phenylpropenal | 1152 | 4432-63-7 | 0.0753 | 0.0832 | 0.1240 | 0.006 | 1.7254 | 0.0009 |
| 23 | 2-Pyridinemethanamine | 1054 | 3731-51-9 | 10.6315 | 11.4152 | 12.1522 | 0.191 | 1.7670 | 0.0022 |
| 24 | 2-Hydroxy-Benzaldehyde | 1047 | 90-02-8 | 0.0665 | 0.1014 | 0.0965 | 0.004 | 1.5309 | 0.0001 |
| 25 | 2-Methoxy-Phenol | 1089 | 90-05-1 | 0.3518 | 0.4071 | 0.4610 | 0.014 | 1.5593 | 0.0073 |
| 26 | (Z)-3-Hexenal | 800 | 6789-80-6 | 2.4260 | 2.6145 | 2.8624 | 0.049 | 1.9396 | 0.0001 |
| 27 | 3-Mercapto-3-Methylbutyl Formate (Ester) | 1023 | 50746-10-6 | 0.2262 | 0.2919 | 0.3056 | 0.011 | 1.5360 | 0.0047 |
| 28 | 4-Methyl-3-Penten-2-One | 798 | 141-79-7 | 3.0668 | 3.2073 | 3.4733 | 0.060 | 1.4927 | 0.0427 |
| 29 | Z-4-Decenoic Acid, Methyl Ester | 1323 | 7367-83-1 | 0.3675 | 0.4968 | 0.7503 | 0.053 | 1.6358 | 0.0037 |
| 30 | 4-Hexen-1-Ol, Acetate | 1020 | 72237-36-6 | 0.2698 | 0.4217 | 0.4431 | 0.033 | 1.8877 | 0.0000 |
| 31 | 5-Methyl-4-Hexen-3-One | 1080 | 13905-10-7 | 0.3782 | 0.4363 | 0.4974 | 0.016 | 1.6170 | 0.0052 |
| 32 | 1,3-Dimethyl-4-Piperidinone | 1065 | 4629-80-5 | 32.6518 | 37.6576 | 39.9270 | 0.790 | 1.9954 | 0.0000 |
| 33 | 4-Methyl-1-(1-Methylethyl)-Bicyclo [3.1.0] Hex-2-Ene | 966 | 28634-89-1 | 1.0653 | 1.1795 | 1.3544 | 0.044 | 1.4700 | 0.0153 |
| 34 | 5,6,7,8-Tetrahydroquinoxaline | 1212 | 34413-35-9 | 0.0603 | 0.0778 | 0.1013 | 0.005 | 1.5182 | 0.0201 |
| 35 | 2,6-Dimethyl-5-Heptenal | 1054 | 106-72-9 | 434.4137 | 476.4210 | 503.9080 | 8.793 | 1.7189 | 0.0021 |
| 36 | 5-Methyloxazolidine | 795 | 58328-22-6 | 21.9506 | 22.9579 | 23.6298 | 0.258 | 1.4313 | 0.0207 |
| 37 | 8-Nonen-2-One | 1085 | 5009-32-5 | 0.3211 | 0.3713 | 0.4410 | 0.016 | 1.6349 | 0.0059 |
| 38 | Acetic Acid, (Propylthio)-, Methyl Ester | 1036 | 20600-65-1 | 0.2892 | 0.3343 | 0.4018 | 0.017 | 1.4504 | 0.0176 |
| 39 | Acetic Acid, Phenyl Ester | 1067 | 122-79-2 | 2.6389 | 2.9092 | 3.0668 | 0.055 | 1.6955 | 0.0022 |
| 40 | 2,4-Dimethyl-Benzaldehyde | 1182 | 15764-16-6 | 0.1293 | 0.1443 | 0.2003 | 0.009 | 1.5316 | 0.0119 |
| 41 | 2,5-Dimethyl-Benzaldehyde | 1208 | 5779-94-2 | 0.0602 | 0.0732 | 0.1012 | 0.005 | 1.5766 | 0.0067 |
| 42 | 1,2,4,5-Tetramethyl-Benzene | 1115 | 95-93-2 | 0.9126 | 1.0434 | 1.3904 | 0.059 | 1.6647 | 0.0026 |
| 43 | 1,4-Dichloro-Benzene | 1021 | 106-46-7 | 0.5702 | 0.6432 | 0.7173 | 0.020 | 1.4918 | 0.0087 |
| 44 | 1-Ethenyl-4-Methoxy-Benzene | 1156 | 637-69-4 | 0.8168 | 0.9149 | 1.3180 | 0.063 | 1.6534 | 0.0029 |
| 45 | 1-Ethyl-3,5-Dimethyl-Benzene | 1059 | 934-74-7 | 0.1824 | 0.2241 | 0.2686 | 0.012 | 1.6440 | 0.0071 |
| 46 | Nitro-Benzene | 1080 | 98-95-3 | 0.2220 | 0.2560 | 0.3333 | 0.012 | 1.9022 | 0.0010 |
| 47 | 4-Methyl-Benzeneacetaldehyde | 1120 | 104-09-6 | 0.0645 | 0.0817 | 0.1043 | 0.005 | 1.6149 | 0.0215 |
| 48 | Alpha-Methyl-Benzenemethanol | 1061 | 98-85-1 | 0.8588 | 0.9721 | 1.0441 | 0.020 | 2.0347 | 0.0001 |
| 49 | Benzothiazole | 1092 | 100-53-8 | 0.2909 | 0.4022 | 0.4255 | 0.024 | 1.2770 | 0.0258 |
| 50 | Benzyl Alcohol | 1036 | 100-51-6 | 1.7702 | 2.0387 | 2.3831 | 0.090 | 1.4574 | 0.0159 |
| 51 | Benzyl Methyl Ketone | 1124 | 103-79-7 | 1.3394 | 1.5237 | 2.0672 | 0.095 | 1.6125 | 0.0048 |
| 52 | Butanoic Acid | 802 | 107-92-6 | 0.2539 | 0.2673 | 0.2970 | 0.007 | 1.3244 | 0.0357 |
| 53 | Carbonochloridodithioic Acid, Methyl Ester | 1033 | 16696-91-6 | 0.2708 | 0.3125 | 0.3725 | 0.016 | 1.3482 | 0.0336 |
| 54 | 1-Methyl-4-(1-Methylethylidene)-Cyclohexene | 1197 | 586-81-2 | 0.3054 | 0.3818 | 0.4023 | 0.018 | 1.3650 | 0.0159 |
| 55 | 4-Methyl-Decane | 1259 | 6117-97-1 | 5.6020 | 5.9851 | 6.1489 | 0.088 | 1.4049 | 0.0252 |
| 56 | 1-(2-Methylphenyl)-Ethanone | 1173 | 577-16-2 | 0.5018 | 0.5556 | 0.7635 | 0.034 | 1.6018 | 0.0039 |
| 57 | 1-(4-Ethylphenyl)-Ethanone | 1277 | 937-30-4 | 0.3643 | 0.4333 | 0.5707 | 0.028 | 1.3844 | 0.0281 |
| 58 | Furaneol | 1070 | 3658-77-3 | 252.6787 | 278.3658 | 294.9199 | 4.973 | 1.8670 | 0.0006 |
| 59 | Hexanoic Acid, 2-Methylpropyl Ester | 1247 | 2601-13-0 | 0.2779 | 0.3257 | 0.3316 | 0.098 | 1.2512 | 0.0309 |
| 60 | 1-Methyl-Indan | 1079 | 767-58-8 | 0.2902 | 0.3385 | 0.3845 | 0.012 | 1.5040 | 0.0130 |
| 61 | Isophorone | 1124 | 78-59-1 | 0.3202 | 0.4089 | 0.6644 | 0.036 | 2.0474 | 0.0000 |
| 62 | Naphthalene | 1182 | 91-20-3 | 15.4947 | 18.3158 | 22.8847 | 0.892 | 1.6161 | 0.0192 |
| 63 | Octane | 800 | 111-65-9 | 14.5687 | 15.6245 | 16.1533 | 0.193 | 1.8156 | 0.0006 |
| 64 | 2-Methyl-Phenol | 1054 | 95-48-7 | 4.0083 | 4.4277 | 4.6628 | 0.076 | 1.9050 | 0.0003 |
| 65 | (2-Methylpropyl)-Pyrazine | 1074 | 29460-92-2 | 0.4371 | 0.5320 | 0.5898 | 0.016 | 2.1612 | 0.0000 |
| 66 | 2,3,4,5-Tetrahydro-6-Propyl-Pyridine | 1028 | 1604-01-9 | 0.2001 | 0.2829 | 0.2901 | 0.012 | 1.6640 | 0.0006 |
| 67 | Quinoline | 1238 | 91-22-5 | 0.2081 | 0.2551 | 0.2551 | 0.009 | 1.1321 | 0.0354 |
| 68 | Trans-Anethole | 1283 | 4180-23-8 | 0.1964 | 0.2215 | 0.3031 | 0.015 | 1.2884 | 0.0461 |
| 69 | 2-Butyl-Thiophene | 1069 | 1455-20-5 | 0.1724 | 0.2094 | 0.2697 | 0.010 | 2.1107 | 0.0000 |
| 70 | Trans-Beta-Ionone | 1486 | 79-77-6 | 86.5638 | 128.7494 | 251.2324 | 2.705 | 1.3928 | 0.0249 |

Abbreviations: VIP, variable importance in projection; SEM, standard error of mean
